# Beneficial Effect of the Traditional Chinese Drug Shu-Xue-Tong on Recovery of Spinal Cord Injury in the Rat

**DOI:** 10.1155/2011/862197

**Published:** 2010-09-08

**Authors:** Li-Yun Jia, An-Hui Yao, Fang Kuang, Yu-Kai Zhang, Xue-Feng Shen, Gong Ju

**Affiliations:** ^1^Institute of Neurosciences, The Fourth Military Medical University, 17 Changlexi Road, Xi'an 710032, China; ^2^Department of Occupational and Environmental Health, Faculty of Military Preventive Medicine, The Fourth Military Medical University, 17 Changlexi Road, Xi'an 710032, China

## Abstract

Shu-Xue-Tong (SXT) is a traditional Chinese drug widely used to ameliorate stagnation of blood flow, such as brain or myocardial infarction. Whether SXT may have therapeutic value for spinal cord injury (SCI), during which ischemia plays an important role in its pathology, remains to be elucidated. We hypothesized that SXT may promote SCI healing by improving spinal cord blood flow (SCBF), and a study was thus designed to explore this possibility. Twenty-five male Sprague-Dawley rats were used. SCI was induced by compression, and SXT was administrated 24 h postinjury for 14 successive days. The effects of SXT were assessed by means of laser-Doppler flowmetry, motor functional analysis (open-field walking and footprint analysis), and histological analysis (hematoxylin-eosin and thionin staining and NeuN immunohistochemistry). SXT significantly promoted SCBF of the contused spinal cord and enhanced the recovery of motor function. Histological analysis indicated that the lesion size was reduced, the pathological changes were ameliorated, and more neurons were preserved. Based on these results we conclude that SXT can effectively improve SCI.

## 1. Introduction

Mechanical trauma of spinal cord causes focal destruction of neural and vascular tissues that is called primary spinal cord injury (SCI), and this initial insult then instigates a progressive wave of secondary SCI [1]. A plethora of factors may contribute to secondary SCI [2–7], among which ischemia plays an important role [8].

The high metabolic demand of spinal cord is met by its abundant blood flow, and changes in its blood supply may result in devastating outcomes. It has been reported that ischemia results in expanding secondary SCI [8, 9], and the degree of ischemia correlates with the severity of injury [10, 11]. A number of studies have been performed targeting at the hypoperfusion after SCI [8, 10, 12–14]. In our previous study, we found that there were two ischemic zones during the secondary SCI, namely, a zone adjacent to the lesion site, where most of the neurons had disappeared or were degenerating, and a farther zone, in which most of the neurons appeared relatively normal [15]. Apparently, there is little chance to rescue the remained severely degenerating neurons in the zone right next to the injury epicenter. However, it is possible to rescue most of the neurons in the farther zone, which is clinically very important. Therefore, improving spinal cord blood flow (SCBF) may well be a measure to ameliorate secondary SCI.

Shu-Xue-Tong (SXT) is a purified extract of leech and earthworm. The main components of SXT are hirudin and lumbrokinase [16, 17]. Hirudin is a specific thrombin inhibitor [18, 19], and lumbrokinase is a strong fibrinolytic enzyme [20]. Both have strong effects on improving blood flow. Thus, SXT has been successfully used to treat patients with cerebral or myocardial infarction [16, 21]. The effect of SXT on SCI has so far not been studied. The present study was aimed to explore its therapeutic value on the secondary SCI. A compression-induced SCI model was used, and the effects were assessed with laser-Doppler flowmetry, open-field walking test, footprint analysis, and histological and immunohistochemistry staining. We found that SXT could significantly ameliorate secondary SCI.

## 2. Methods

### 2.1. Animals

Twenty-five male Sprague-Dawley rats (200–220 g, provided by the Animal Center of the Fourth Military Medical University) were randomly divided into three groups. (1) SCBF assay group: 4 each for saline control and SXT treatment. (2) Motor behavior analysis group: 5 for sham operation, 6 each for saline control and SXT treatment. (3) Morphological analysis group: 6 each for saline control and SXT treatment. All the rats were maintained on a 12/12 h light/dark cycle and were allowed free access to food and water. The procedures were reviewed and approved by the Animal Care Committee of the Fourth Military Medical University. All efforts were made to minimize the number of animals used and their suffering. At the end of the studies, all rats were euthanized with overdose of sodium pentobarbital.

### 2.2. Surgical Procedure

The rats were anesthetized with 1% sodium pentobarbital (50 mg/kg) i.p. A 30–40 mm dorsal midline incision was made, and bilateral laminectomy was performed at T8 level (corresponding to T9 segment of the spinal cord). According to the transverse diameter of the spinal cord we designed a plastic spinal cord compression plate with a thickness of 0.5 mm and a breadth of 2.8 mm. The compression plate was attached to a copper rod with a total weight of 20 g. The injury of the spinal cord was produced by applying the compression plate on the dura mater perpendicular to the longitudinal axis of spinal cord. The compression plate was then lowered down ventrally at a rate of 0.5 mm/min to the bottom of the vertebral canal and remained there for 5 min. After withdrawal of the compression plate, the skin incision was closed. The operated animals were kept in cages with soft bedding, and the ambient temperature was maintained at 20–23°C. Manual evacuation of urinary bladder was performed twice daily until an autonomous micturition reflex developed. In the sham group, only bilateral laminectomy was performed.

### 2.3. Drug Administration

0.25 mL/kg bodyweight per day of SXT (MuDanJiang Youbo Pharmaceutical Co. Ltd., China) was administered intravenously, according to Jin et al. [22]. The time to start the treatment was carefully estimated. The use of SXT, which contains a thrombin inhibitor and a fibrinolytic enzyme, may cause additional bleeding at acute hemorrhagic phase of SCI. The bleeding should have stopped before 24 h. We did pilot experiment to examine if the use of SXT at 24 h after injury might induce fresh bleeding, which turned out to be not the case. Therefore, the first injection of SXT was started at 24 h after injury, and thereafter for 14 successive days. For saline controls, the same amount of saline instead of SXT was administered.

### 2.4. Laser-Doppler Flowmetry

The SCBF was measured using a MoorLab laser-Doppler flowmeter (wavelength, 780 nm) and a MP4 probe (Moorlab Instruments, Devon, England) with the afferent and efferent fibers separated by 0.25 mm. The laminectomized animals from the saline and SXT groups were mounted onto a stereotactic instrument equipped with vertebra fixation device to stabilize the vertebral column. Body temperature was kept at 37.0 ± 0.5°C with a heating blanket (Animal Regulator, BME-421A, Institute of Biomedical Engineering, Tianjin, China). The laser-Doppler probe was affixed to a micromanipulator and placed perpendicularly to the spinal cord, barely touching the dorsal surface of the dura mater. The readings of the laser-Doppler signals were recorded into a computer and analyzed with Moorsoft for Windows V 1.31. SCBF was measured before SCI (0 h) and at 0.5 h, 3 d, and 7 d after SCI in both saline and SXT groups. The measurement taken before SCI was used as the normal control and that taken at 0.5 h was used to examine SCBF right after the injury. For each later measurement, the dura mater should be exposed, which would aggravate the adhesion between the dura mater and the adjacent tissue. Therefore, SCBF measuring would become impossible after 7 d. Four areas with minimal pial vessels were chosen to measure SCBF, two points on each side at 1 mm rostral and caudal to the lesion epicenter. The value for each area was taken as the mean of consecutive spikes obtained from 1 min recording. The final value for each rat was obtained from the average value of the four areas. The mean value of the four regions before injury (0 h) was taken as the basal value, and the final value for each rat at each later time point was expressed in percentage of the basal value (100%).

### 2.5. Functional Assessment

#### 2.5.1. Open-Field Walking Assessment

Motor functional recovery was evaluated at 7 d and 14 d using the open-field walking scoring system. The hindlimb movements were scored according to the Tarlov's motor scale: 0, no spontaneous movement; 1, movement in the hip and/or knee but not ankle; 2, movement of the limb in all three major joints; 3, active support, uncoordinated gait; 4, coordination of forelimbs and hindlimbs in gait; 5, normal gait and base of support, no loss of balance on fast turns, and no toe dragging [23].

#### 2.5.2. Footprint Analysis

The analysis was conducted at 7 d and 14 d. The plantar surface of both hindlimbs of each rat was colored black and the dorsal surface red with nontoxic inks. After that, the rat was allowed to run towards a dark tunnel on the white paper (21 × 59.4 cm^2^) [24]; thus the red and/or black ink was printed onto the paper as one set of footprints. From each set of footprints, the pixels of the black (normal plantar paw placing) and red (abnormal dorsal paw placing) areas were determined using Adobe Photoshop 9.0. The percentage of red pixels defined as red/(black + red) × 100% was computed to quantify toe dragging. Stride length (distance between the centers of ipsilateral adjacent footprints) and stride width (perpendicular distance between the centers of left and right hind limbs) were measured, and the average of five steps in each case was used for statistical comparison.

### 2.6. Morphological Studies

At 14 d, after footprint analysis, the animals were sacrificed by an overdose of sodium pentobarbital (100 mg/kg) and perfused intracardially with saline (37°C) followed by 400 mL 4% cold paraformaldehyde phosphate buffer (pH 7.4). Following perfusion, a 20 mm spinal cord segment with the injured site at its center was removed and put into 25% sucrose in phosphate buffer at 4°C until the block sank. Serial 20 *μ*m frozen sagittal sections were cut on a cryostat and mounted on slides in 10 sets. Hematoxylin and eosin (HE) staining, thionin staining, and NeuN immunostaining were performed, respectively, each on one of the 10 sets. For immunostaining, the sections were rinsed with 0.01 M phosphate buffer saline (PBS) and then blocked with 1% bovine serum albumin (Sigma) in PBS containing 0.3% Triton X-100 for 1 h at room temperature. Sections were then incubated with anti-NeuN antibody (1 : 1000; Chemicon, Temecula, CA) for 48 h at 4°C. After washing with PBS, the NeuN immunoreactivity was detected by incubating with Alexa Fluor 594 donkey antimouse IgG (1 : 800; Molecular Probes, Oregon, USA) for 24 h at 4°C. Omission of the primary antibody served as the negative control. The sections were observed under an Olympus BX-51 microscopy.

### 2.7. Quantification and Statistical Analysis

#### 2.7.1. Area of Injury

HE stained sections were used for analysis of the area of the injury. Five sections were chosen for analysis in each rat, namely, the section with central canal and two adjacent sections on both sides, with 200 *μ*m between each section. The microscopic images were obtained with a 40x objective under an Olympus BX-51 microscopy. The images were opened in Adobe Photoshop 9.0 and stuck together as a montage. The boundary of the injured area was outlined according to the differences between normal tissue and necrotic or apoptotic tissue and captured with the noose tool. The pixel of the outlined area was calculated and then converted to the size of injury.

#### 2.7.2. Count of Neurons

Five sections similar to those mentioned above were chosen for analysis in each rat. The number of NeuN positive cells within 1000 *μ*m distance to the injured border was counted with a 200x objective under an Olympus BX-51 microscopy. The mean values for the five sections in each rat were used for statistical analysis.

#### 2.7.3. Statistical Analysis

All data were expressed as mean ± SD. Values of open-field walking activity were analyzed by the nonparametric Mann-Whitney *U* test. All the other results were compared with one-way analysis of variance (ANOVA). The statistical program SPSS 12.0 for windows was used for statistic analysis. *P* < .05 was considered significant.

## 3. Results

### 3.1. Compression-Induced SCI Model

The pial vasculature on the dorsal surface could be clearly identified in the normal spinal cord. After SCI, a transverse hemorrhagic band resulting from compression could be observed on the dorsal surface. HE-stained sagittal sections showed that severe hemorrhage occurred at the lesion epicenter immediately after injury. The compression also caused some bleeding in parts rostral and caudal to the site of compression.

### 3.2. Spinal Cord Blood Flow

Saline control: laser-Doppler flowmetry assay showed that the SCBF was 58 ± 6.20% at 0.5 h after SCI, 65 ± 3.81% at 3 d, and 61 ± 6.68% at 7 d. Statistic analysis showed no significant difference among them (*P* > .05).

SXT Group: the SCBF value increased from 57 ± 6.43% at 0.5 h to 78 ± 4.16% at 3 d (*P* < .01) and further increased to 84 ± 2.20% at 7 d (*P* < .05).

### 3.3. Analysis of Motor Function

Any operation will affect the behavior of the animal. Therefore, for behavioral assessment, a sham group, in which the animals simply receive bilateral laminectomy, was included.

#### 3.3.1. Open-Field Walking Test

It has been proved in the rat that functional recovery could be achieved significantly if as few as 5%–10% white matter was spared [25]. Therefore, in the open-field walking test, we compared the motor functional recovery at the corresponding time points between injured groups, rather than comparing at different time points within the same group. All the rats showed normal hindlimb movements (scored 5) before injury. Immediately after injury, the rats in both injured groups showed complete paraplegia (scored 0). However, by 7 d, the SXT group recovered considerable bilateral motor function with a score of 2.83 ± 0.41, allowing occasional body weight support, while the saline group only showed extensive movement of hip or knee with no stepping (1.62 ± 0.52, *P* < .01, compared with SXT group). By 14 d, the SXT group showed a close hindlimb and forelimb coordination (3.83 ± 0.41). In contrast, the saline group showed a score of 2.67 ± 0.52 (*P* < .01, compared with SXT group) with only occasional weight support but no stepping. The sham group showed normal stepping.

#### 3.3.2. Footprint Analysis

**Figure 1:**
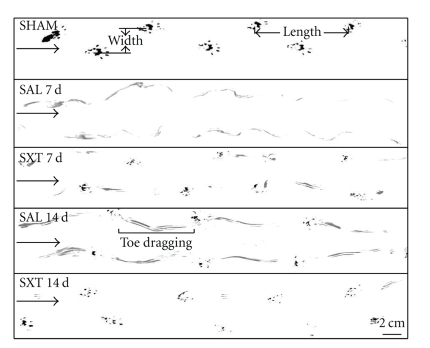
Footprint analysis at 7 and 14 days after injury. Arrows point to the direction of walking. SHAM: sham group; SAL: saline control; SXT: SXT treated group. SHAM: normal walking with normal stride width and length. SAL 7 d: there is only toe dragging. No plantar stepping appears. SXT 7 d: plantar prints appear. SAL 14 d: plantar stepping begins to appear in the saline group. The toe dragging was still serious. SXT 14 d: the animal resumes almost normal walking. Scale  bar = 2 cm.

The SCI in this study resulted in toe dragging, decrease in stride length, and increase in stride width. Representative running tracings from each treatment were shown in Figure 1. The sham group showed consistent normal plantar stepping during the whole process. At 7 d, severe toe dragging with no planar stepping was observed in the saline group, while the SXT group began to show planar footprints. At 14 d, in the saline group the toe dragging was still serious, and the plantar stepping began to appear, while the SXT group showed improved plantar stepping with a little dorsum prints remaining. In all the time points with plantar stepping, the stride length increased, and the stride width decreased progressively. Also improved were the planter prints, but none was quite complete as compared to the sham group.

**Figure 2:**
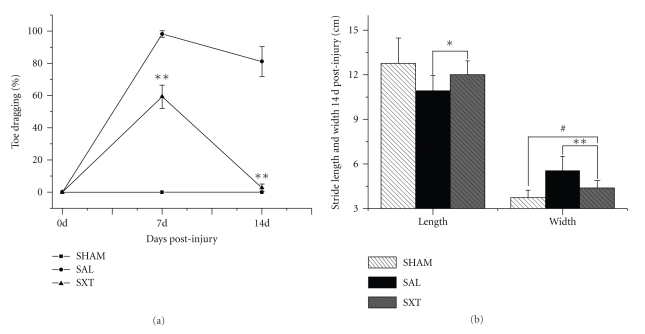
Statistical analysis of footprints. SHAM: Bilaterally laminectomized. 0 d: Preinjury normal control. SAL: saline control, SXT: Shu-Xue-Tong treated. (a) The SAL group shows no recovery at 7 d, 14 d, while the SHAM group has no toe dragging at all. The SXT group resumes significant plantar stepping at 7 d and is almost normal at 14 d. (b) Both stride width and stride length are significantly improved in the SXT group at 14 d. **P < .01; *P < .05; ^#^ *P* < .05.

Quantitative analysis showed that compared with the saline group, the toe dragging in the SXT group was significantly ameliorated at 7 d and 14 d (59.33 ± 7.23% versus 98.22 ± 1.99% at 7 d; 2.71 ± 2.43% versus 81.14 ± 9.19% at 14 d; *P* < .01) (Figure 2(a)). Because of severe toe dragging with no plantar stepping at 7 d, the stride length and width analysis were only possible in 14 d studies. Compared with the saline group the stride length in the SXT group was markedly increased (10.93 ± 1.02 cm versus 12.01 ± 0.93 cm; *P* < .05) and the stride width significantly reduced (5.58 ± 0.95 cm versus 4.39 ± 0.49 cm; *P* < .01) (Figure 2(b)). Furthermore, in all the experimental groups the hind limb plantar stepping was laterally rotated.

### 3.4. Histological Study

#### 3.4.1. HE and Thionin Staining

**Figure 3:**
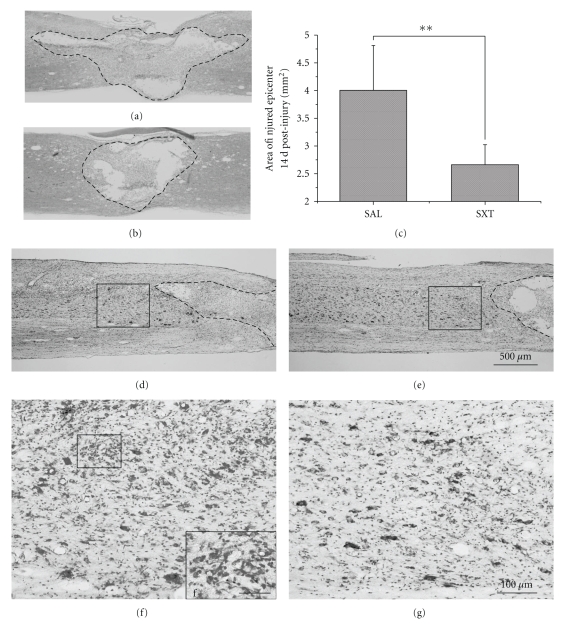
HE and thionin staining at 14 d. (a) and (b) HE staining. (a) Saline control, (b) SXT treated. The boundary of the lesion site is outlined. The lesion area is significantly smaller in (b). (c) The lesion area is prominently reduced in the SXT group (*P* < .01). (d)–(g) Thionin staining. (d) and (f) Saline control. (e) and (g) SXT treated. The boxes in (d), (e) are the areas adjacent to the lesion sites, showing that there is larger number of neurons in the SXT treated animal. (f) and (g) are successive blown-ups of the boxes in (d) and (e), displaying the abundance of microglia cells in the saline control. (f in (f) is the high magnification of the box at the upper left part of the figure). Scale bar in (a), (b) and (d)–(e) = 500 *μ*m; in (f) and (g) = 100 *μ*m; in (f) = 25 *μ*m.

On the 14 d HE-stained sections, the saline group showed extensive necrosis at the injury site with prominent cavitation (Figure 3(a)). In the SXT-treated group the lesion size was significantly smaller, and the cavitation was milder (Figure 3(b)). The area of the lesion was 4.01 ± 0.81 mm^2^ in the saline group and 2.67 ± 0.36 mm^2^ in the SXT group (*P* < .01). The histopathological changes displayed in the thionin-stained sections demonstrated that the SXT group showed more neurons and less inflammatory cells in the area adjacent to the lesion site compared with the saline group (Figures 3(d)–3(g)).

#### 3.4.2. NeuN Immunohistochemistry

**Figure 4:**
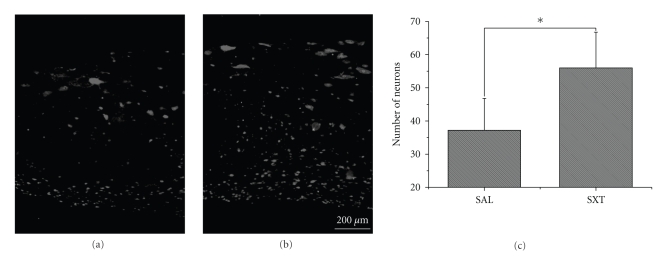
NeuN immunohistochemistry at 14 d. (a) Saline control. (b) SXT treated. The photos of two areas of similar size were taken 1000 *μ*m away from the lesion. Note that there is larger number of neurons in the SXT group. Scale bar = 200 *μ*m. (c) As compared to the SAL group, the number of neurons is significantly higher in the SXT group (*P* < .05).

The number of NeuN-immunoreactive neurons in an area 1000 *μ*m away from the edge of the lesion was counted. Compared to the saline group (37.2 ± 9.60), a significantly larger number of neurons were present in the SXT group (56 ± 10.79, *P* < .05) (Figures 4(a)–4(c)).

**Figure 5:**
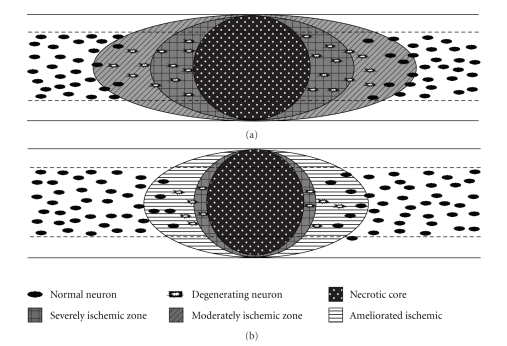
Diagrammatic summary of the beneficial effect of SXT in ameliorating the secondary injury.

The beneficial effect of SXT in ameliorating the secondary injury is summarized in Figure 5.

## 4. Discussion

The main causes of SCI are traffic accidents, fall from a hight, and weight crushing. In many cases the fracture or dislocation of vertebral column results in compression or hyperextension of the spinal cord [26], resulting in paralysis of limbs. The spinal cord is rarely totally transected even after severe contusive injury associated with complete paralysis [5, 27]. It has been proved that sparing as few as 5%–10% of the fibers in the spinal cord is sufficient to facilitate basic locomotion recovery following SCI injury in rats [25]. That the majority of SCI are incomplete and that a small percentage of spared fibers are sufficient for resumption of basic locomotion encourage researches for the treatment of SCI [28–30].

A major clinical concern is to minimize secondary degeneration following SCI. During the process of secondary SCI, there exists a penumbral ischemic zone adjacent to the lesion epicenter, and this region exhibits progressive cell death over time [8]. We found two zones next to the injury epicenter with different degrees of ischemia, which brings us hope that neurons in the farther zone can be rescued by enhancing the SCBF [15]. In this study SXT was used to promote blood flow in the spinal cord. It has been widely used in China to treat patients suffering from various infarctions, such as cerebral or myocardial infarction [16, 21]. The main effect of SXT is to improve blood flow through its two components, namely, hirudin and lumbrokinase [17]. Our results demonstrated that SXT promoted SCBF in crushed spinal cord, facilitated motor functional recovery, decreased the lesion size, and rescued neurons in the ischemic area near the lesion site. One of the beneficial effects of acupuncture in treating spinal cord injuries has been reported to be the increase in SCBF [31, 32]. It lends support to our present study. Moreover, it has been reported that one of the major components of SXT, hirudin, being a specific thrombin inhibitor, is able to reduce thrombin-induced brain edema [18, 19]. Thus, it may well be possible that the beneficial effect of SXT on functional recovery in SCI is in part credited to the reduction of spinal cord edema.

In conclusion, the present study demonstrated that SXT can significantly promote SCBF, minimize secondary injury, protect neurons in the ischemic area, and facilitate motor recovery. These findings laid a basis for future clinical application of SXT for the treatment of SCI.
